# Supplementary material for: Aptamer-Based Proteomics Measuring Preoperative Cerebrospinal Fluid Protein Alterations Associated with Postoperative Delirium
Source: Biomolecules. 2023 Sep 15;13(9):1395. doi: 10.3390/biom13091395 (PMC10526755; doi:10.3390/biom13091395)

Supplementary Methods:

Assessment of Delirium and Severity in HiPOR

Diagnosis of delirium was performed using the Confusion Assessment Method (CAM) diagnostic algorithm that incorporates the presence of acute change/fluctuating course, inattention, and either disorganized thinking or an altered level of consciousness [48]. The CAM is a standardized and widely employed approach to assess the presence or absence of delirium with high sensitivity (94-100%) and specificity (90-95%) and high inter-rater reliability (kappa = 0.92) [49]. Subsyndromal delirium (SSD) was also defined from the CAM, and required the presence of acute change/fluctuating course, and at least one other feature of delirium, but did not fulfill the full diagnostic criteria for delirium described above. Delirium was determined to be present if CAM criteria were fulfilled on POD1, 2, or 3. SSD was present if the patient never met CAM criteria for delirium, but the patient met criteria for SSD on POD 1, 2, or 3. If none of these criteria were met, then non-delirium was considered to be present. SSD patients were excluded from the available pool of samples for the match since they were neither cases or controls.

Days of delirium was employed as a proxy for severity. Delirium lasting only one day was scored as mild while delirium lasting ≥2 days was considered severe. Spearman's correlation analysis of the unmatched proteomics data was used and identified 23 proteins significantly associated with delirium severity. None of these proteins demonstrated a significant Benjamini-Hochberg (BH)-correction ($p<0.05$).

Proteomics Analysis Using SomaScan

CSF samples were run on the SomaScan Assay Cells & Tissue Kit, 1.3k (SomaLogic #900-00009, SomaLogic, Inc., Boulder, CO USA), which measures the expression of 1305 human proteins using single-stranded modified Slow Off-rate Modified DNA Aptamers (SOMAmers) [50]. 20 ul of CSF from each patient sample were run at a 15% dilution in 1X serum diluent. Three provided kit controls and one no-protein buffer control were analyzed in parallel with the CSF samples. Median normalization and calibration of the SomaScan data were performed according to standard quality control (QC) protocols at SomaLogic [51]. All 48 HiPOR samples, plus all controls, passed the QC criteria.

Molecular and Systems Biology Analysis

The consistency of findings across multiple analysis algorithms results in higher confidence of an individual protein's function and its reported biological pathways [52]. IPA generated functional category, canonical pathway, interactive network, and upstream regulator analysis of the proteins with a paired t-test $p<0.05$. IPA contains a regularly updated and curated repository of biological interactions and functions created from millions of individually modeled relationships ranging from molecular (proteins, genes) to organism (diseases) level. This software leverages enrichment analysis-based approaches to calculate the significance of observing a candidate protein set within the context of biological systems.

The 32 dysregulated proteins with a $p<0.05$ were included in network analysis by applying the Search Tool for the Retrieval of Interacting Genes/Proteins (STRING) database [53]. The STRING database version 11.0 was employed for protein-protein functional and physical interactions, and these were displayed as a functional network. Interactions were considered with a STRING confidence score of 0.4 or higher garnered from the "experimental"

and “databases” categories. Proteins without associations to other proteins in the network were removed. A k-means clustering algorithm was performed to select connected proteins (k-means = 5). Functional description of clusters was assigned based on a manually curated evaluation of enriched KEGG pathway, GO, Reactome, STRING local network clusters terms, and PubMed literature search.

References:

48. Inouye SK, van Dyck CH, Alessi CA, Balkin S, Siegal AP, Horwitz RI. Clarifying confusion: the Confusion Assessment Method. A new method for detection of delirium. Ann Intern Med. 1990;113:941–8.
49. Inouye SK, Marcantonio ER, Kosar CM, Tommet D, Schmitt EM, Travison TG, et al. The short-term and long-term relationship between delirium and cognitive trajectory in older surgical patients. Alzheimers Dement. 2016;12:766-75.
50. Gold L, Walker JJ, Wilcox SK, Williams S. Advances in human proteomics at high scale with the SomaScan proteomics platform. N Biotechnol. 2012;29:543-9.
51. Candia J, Cheung F, Kotliarov Y, Fantoni G, Sellers B, Griesman T, et al. Assessment of Variability in the SomaScan Assay. Sci Rep. 2017;7:14248-61.
52. Mubeen S, Hoyt CT, Gemünd A, Hofmann-Apitius M, Fröhlich H, Domingo-Fernández D. The Impact of Pathway Database Choice on Statistical Enrichment Analysis and Predictive Modeling. Front Genet. 2019;10:1203-16.
53. Szklarczyk D, Gable AL, Lyon D, Junge A, Wyder S, Huerta-Cepas J, et al. STRING v11: protein-protein association networks with increased coverage, supporting functional discovery in genome-wide experimental datasets. Nucleic Acids Res. 2019;47:D607-13.

Supplementary Table S1: Catalog and Distribution of Co-morbidities Present in the 48 HiPOR Patients Selected for Proteomics Analysis

Medical condition	controls (n = 24)*	cases (n = 24)*
acute myocardial infarction	0 (0%)	1 (4%)
chronic heart disease	0 (0%)	0 (0%)
peripheral vascular disease	0 (0%)	1 (4%)
cerebrovascular disease	0 (0%)	0 (0%)
dementia	0 (0%)	0 (0%)
chronic pulmonary disease	0 (0%)	0 (0%)
rheumatoid disease	1 (4%)	0 (0%)
peptic ulcer disease	0 (0%)	0 (0%)
mild liver disease	0 (0%)	0 (0%)
moderate liver disease	0 (0%)	0 (0%)
diabetes with complications	2 (8%)	2 (8%)
diabetes without complications	0 (0%)	0 (0%)
hemiplegia	0 (0%)	0 (0%)
renal disease	0 (0%)	0 (0%)
history of cancer#	0 (0%)	5 (21%)
metastatic tumor	0 (0%)	0 (0%)
AIDS (HIV)	0 (0%)	0 (0%)

*number of patients with condition (% with condition)
#bladder (1), breast (1), colon (1), prostate (3)
one patient had a history of both bladder and prostate

Supplementary Table S2: Significant Upregulated and Downregulated Delirium Incidence Proteins Associated with Delirium Severity

**Increased in Delirium Incidence and Severity**				**incidence**	**severity**
**Full Name of Protein**	**UniProt**	**Gene Symbol**	**tFC**	**t-test p-val**	**S p-val**
Aggrecan core protein**	P16112	ACAN	1.79	0.013	0.007
Histone H2A.z**	P0C0S5	H2AFZ	1.44	0.020	0.021
Nicotinamide phosphoribosyltransferase**	P43490	NAMPT	1.36	0.029	0.013
Insulin**	P01308	INS	1.35	0.019	0.028
Inducible T-cell costimulator**	Q9Y6W8	ICOS	1.31	0.028	0.045
Lymphotactin	P47992	XCL1	1.30	0.169	0.040
Tyrosine-protein kinase ZAP-70	P43403	ZAP70	1.29	0.076	0.014
Cerebral dopamine neurotrophic factor	Q49AH0	CDNF	1.23	0.061	0.011
beta-nerve growth factor**	P01138	NGF	1.20	0.020	0.003
MHC class I polypeptide-related sequence A**	Q29983	MICA	0.90	0.011	0.031
**Decreased in Delirium Incidence and Severity**					
Kallikrein-5	Q9Y337	KLK5	-1.38	0.095	0.028
C-X-C motif chemokine 6**	P80162	CXCL6	-1.36	0.017	0.047
RAC-beta serine/threonine-protein kinase	P31751	AKT2	-1.32	0.163	0.023
Sorting nexin-4	O95219	SNX4	-1.31	0.112	0.048
Moesin**	P26038	MSN	-1.24	0.014	0.007
C-C motif chemokine 28**	Q9NRJ3	CCL28	-1.21	0.015	0.017
Copine-1	Q99829	CPNE1	-1.19	0.087	0.025
Cathepsin D**	P07339	CTSD	-1.15	0.021	0.049
Follistatin-related protein 1**	Q12841	FSTL1	-1.12	0.040	0.038
Insulin-like growth factor-binding protein 2**	P18065	IGFBP2	-1.07	0.028	0.043
Prostate-specific antigen	P07288	KLK3	-1.06	0.080	0.036
Tyrosine-protein phosphatase non-receptor type 6**	P29350	PTPN6	-1.04	0.044	0.044
Interstitial collagenase	P03956	MMP1	-0.94	0.107	0.050

tFC = Tukey Fold Change, S = Spearman
**significant for both delirium incidence (t-test ($p<0.05$)) and severity (Spearmen ($p<0.05$))

Supplementary Table S3: Combined List of Unique Upregulated and Downregulated Proteins Analyzed for Delirium Incidence and Delirium Severity

Increased in Delirium Incidence and Severity				incidence	severity
**Full Name of Protein**	**UniProt**	**Gene Symbol**	**tFC**	**t-test p-val**	**S p-val**
Aggrecan core protein**	P16112	ACAN	1.79	0.013	0.007
Cofilin-1*	P23528	CFL1	1.62	0.041	0.060
C-X-C motif chemokine 11	O14625	CXCL11	1.46	0.047	0.396
Histone H2A.z**	P0C0S5	H2AFZ	1.44	0.02	0.021
Mucin-1*	P15941	MUC1	1.39	0.039	0.090
Nicotinamide phosphoribosyltransferase**	P43490	NAMPT	1.36	0.029	0.013
Insulin**	P01308	INS	1.35	0.019	0.028
CD97 antigen	P48960	CD97	1.32	0.006	0.134
Inducible T-cell costimulator**	Q9Y6W8	ICOS	1.31	0.028	0.045
Protein deglycase DJ-1	Q99497	PARK7	1.31	0.03	0.117
Lymphotactin	P47992	XCL1	1.30	0.169	0.040
Protein FAM107B*	Q9H098	FAM107B	1.29	0.028	0.063
ADP-ribosyl cyclase/cyclic ADP-ribose hydrolase 1*	P28907	CD38	1.29	0.048	0.082
Tyrosine-protein kinase ZAP-70#	P43403	ZAP70	1.29	0.076	0.014
Cerebral dopamine neurotrophic factor#	Q49AH0	CDNF	1.23	0.061	0.011
beta-nerve growth factor**	P01138	NGF	1.2	0.02	0.003
Peptidyl-prolyl cis-trans isomerase F, mitochondrial*	P30405	PPIF	1.17	0.035	0.051
Thrombopoietin*	P40225	THPO	1.12	0.01	0.075
Decorin	P07585	DCN	1.09	0.013	0.120
MHC class I polypeptide-related sequence A**	Q29983	MICA	0.9	0.011	0.031
Hyaluronan and proteoglycan link protein 1	P10915	HAPLN1	0.86	0.042	0.167
**Decreased in Delirium Incidence and Severity**					
Cathepsin L2*	O60911	CTSV	-1.47	0.048	0.053
Granulysin	P22749	GNLY	-1.43	0.045	0.231
Kallikrein-5#	Q9Y337	KLK5	-1.38	0.095	0.028
C-X-C motif chemokine 6**	P80162	CXCL6	-1.36	0.017	0.047
Sorting nexin-4	O95219	SNX4	-1.31	0.112	0.048
C-C motif chemokine 2*	P13500	CCL2	-1.34	0.03	0.070
RAC-beta serine/threonine-protein kinase	P31751	AKT2	-1.32	0.163	0.023
Matrix metalloproteinase-14*	P50281	MMP14	-1.29	0.03	0.077
Moesin**	P26038	MSN	-1.24	0.014	0.007
Chordin-like protein 1*	Q9BU40	CHRDL1	-1.22	0.035	0.095
Activated Protein C*	P04070	PROC	-1.21	0.015	0.061
C-C motif chemokine 28**	Q9NRJ3	CCL28	-1.21	0.015	0.017
Copine-1#	Q99829	CPNE1	-1.19	0.087	0.025
Cathepsin D**	P07339	CTSD	-1.15	0.021	0.049
Follistatin-related protein 1**	Q12841	FSTL1	-1.12	0.04	0.038
Insulin-like growth factor-binding protein 2**	P18065	IGFBP2	-1.07	0.028	0.043
Prostate-specific antigen#	P07288	KLK3	-1.06	0.080	0.036
Tyrosine-protein phosphatase non-receptor type 6**	P29350	PTPN6	-1.04	0.044	0.044
Protein kinase C alpha type#	P17252	PRKCA	-1.02	0.035	0.086
Interstitial collagenase	P03956	MMP1	-0.94	0.107	0.050

tFC = Tukey Fold-Change, S = Spearman

*significant by t-test (p<0.05) for delirum and severity by Spearmen (p<0.10)

#t-test (p<0.10) for delirium and significant for severity by Spearman (p<0.05))

**significant by both t-test (p<0.05) for delirium and for severity by Spearmen (p<0.05)

Supplementary Figure Legends:

Supplementary Figure S1: Biological Pathway Analysis of 32 CSF Proteins Associated with Delirium

Systems biology analysis by IPA of the 32 significant differentially expressed proteins. (A). Inflammatory response. (B). Migration/chemotaxis of myeloid cells. All myeloid cell movement pathways are presented in the diagram. (C). Apoptosis. (D). Long-term synaptic depression. (E). Angiogenesis. (F). Neuronal cell death. IPA shape and color code: red indicates upregulation, green denotes downregulation. Proteins are coded by shape: square, cytokine; vertical rhombus; enzyme, horizontal rhombus; peptidase, trapezoid; transporter, ellipse; transmembrane receptor, circle; other, rectangle. Lines are color-coded: red, leads to activation; blue, leads to inhibition; yellow, findings are inconsistent with the status of the downstream protein; black, effect not predicted. For pathway hubs represented in the wheel diagrams orange indicates predicted activation; blue represents predicted inhibition. The relative color shade is related to the level of confidence.

Supplementary Figure S2: Upstream Regulator Analysis of the 32 Significant CSF Proteins

Ingenuity pathway analysis (IPA) was used to determine upstream regulatory proteins that affect the identified 32 significant proteins. Upstream regulators presented in wheel diagrams in Supplementary Figure 3 are boxed in red. Level of significance: $p<10^{-5}$.

Supplementary Figure S3: Proteins Differentially Expressed in Delirium for Cytokine and Chemokine Signaling Pathways

Wheel diagrams depicting interactions of each regulator with relevant proteins from the CSF discriminatory panel. The hub is the cytokine and chemokine regulator. (A). Cytokine tumor necrosis factor-α; activated. (B). Cytokine interferon-γ; activated. (C). Cytokine interleukin-6; activated. (D). Cytokine interleukin-10; activated. (E.) Cytokine transforming growth factor-β1; inhibited. IPA shape and color code: red indicates upregulation, green denotes downregulation. Proteins are coded by shape: square, cytokine; vertical rhombus; enzyme, horizontal rhombus; peptidase, trapezoid; transporter, ellipse; transmembrane receptor, circle; other, rectangle. Lines are color-coded: red, leads to activation; blue, leads to inhibition; yellow, findings inconsistent with status of downstream protein; black, effect not predicted. For pathway hubs represented in the wheel diagrams orange indicates predicted activation; blue represents predicted inhibition. The relative darkness of the color shade is related to the level of confidence.

Supplementary Figure S4: Shared Overlap of Proteins for Delirium Incidence and Delirium Severity

Venn diagram analysis illustrating the overall number of shared proteins between delirium incidence and delirium severity at different levels of significance or near significance. (A). Delirium incidence t-test p-value < 0.05 and delirium severity Spearman p-value < 0.05. Fourteen proteins out of the unique total of 41 proteins are in common. (B). Delirium incidence

t-test p-value < 0.10 and delirium severity Spearman p-value < 0.10. Thirty-one proteins out of the unique total of 41 proteins are in common.

Supplementary Figure S1: Significant Biological Pathways for Preoperative CSF Proteins in Delirium

A. Inflammatory response

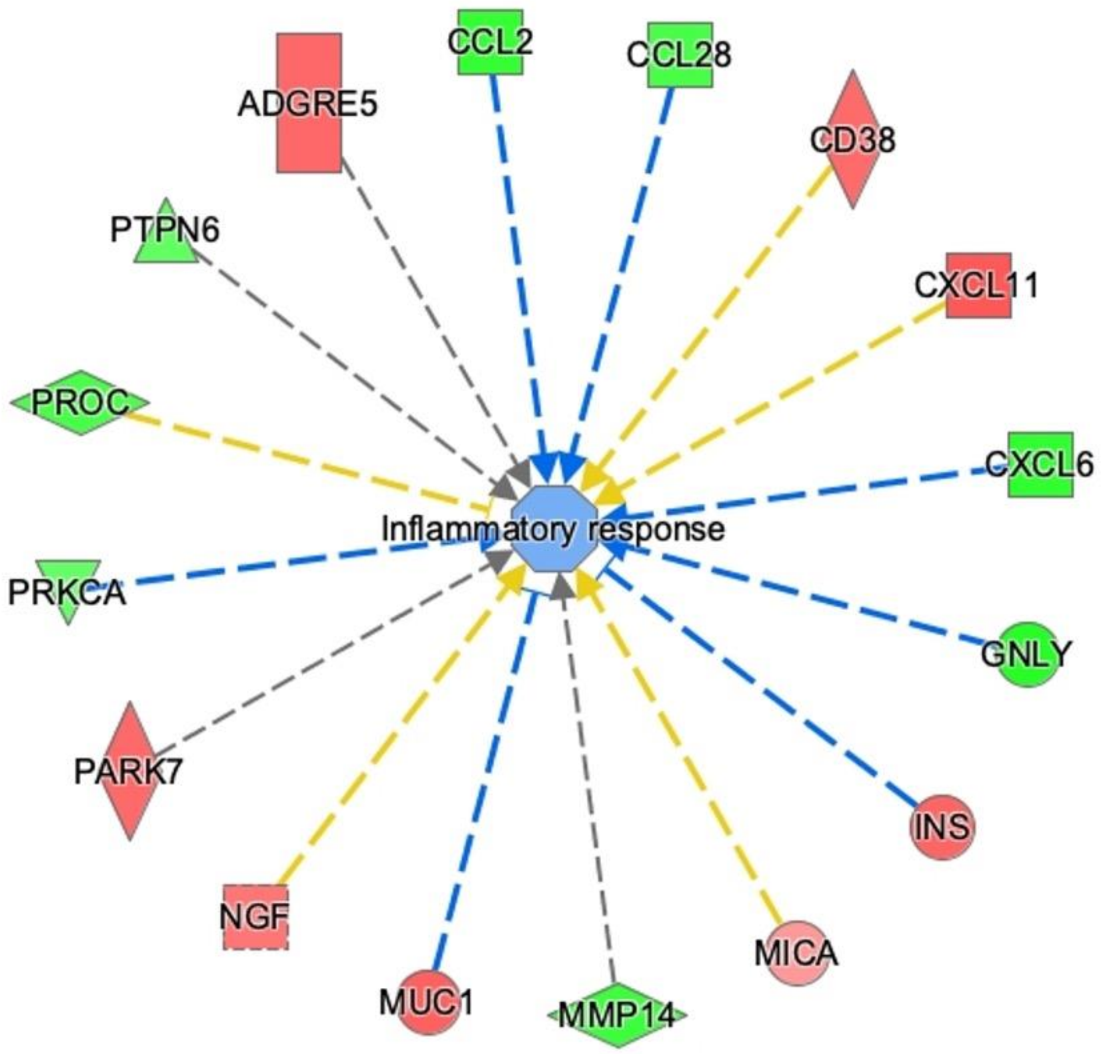

B. Immune cell migration/chemotaxis

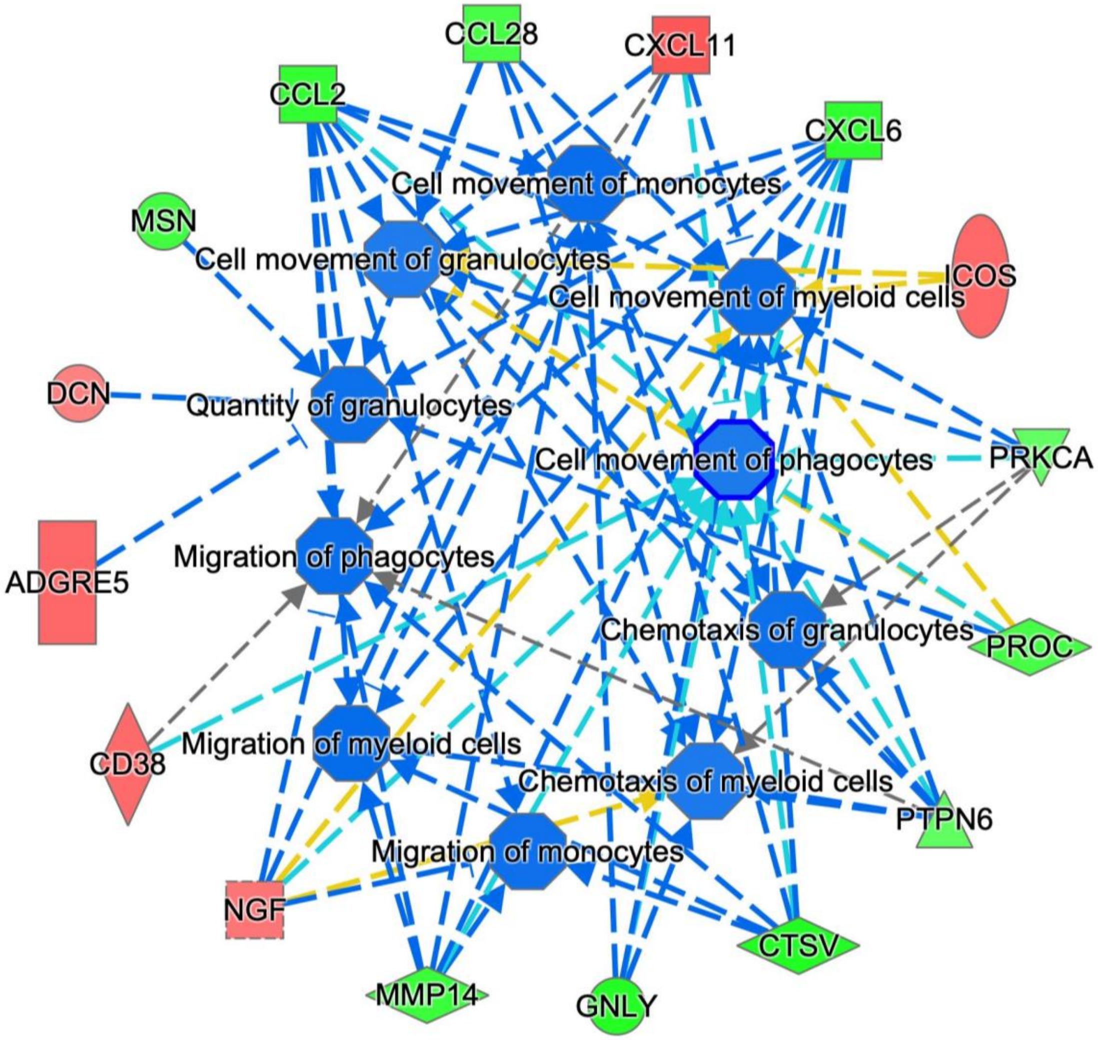

C. Apoptosis

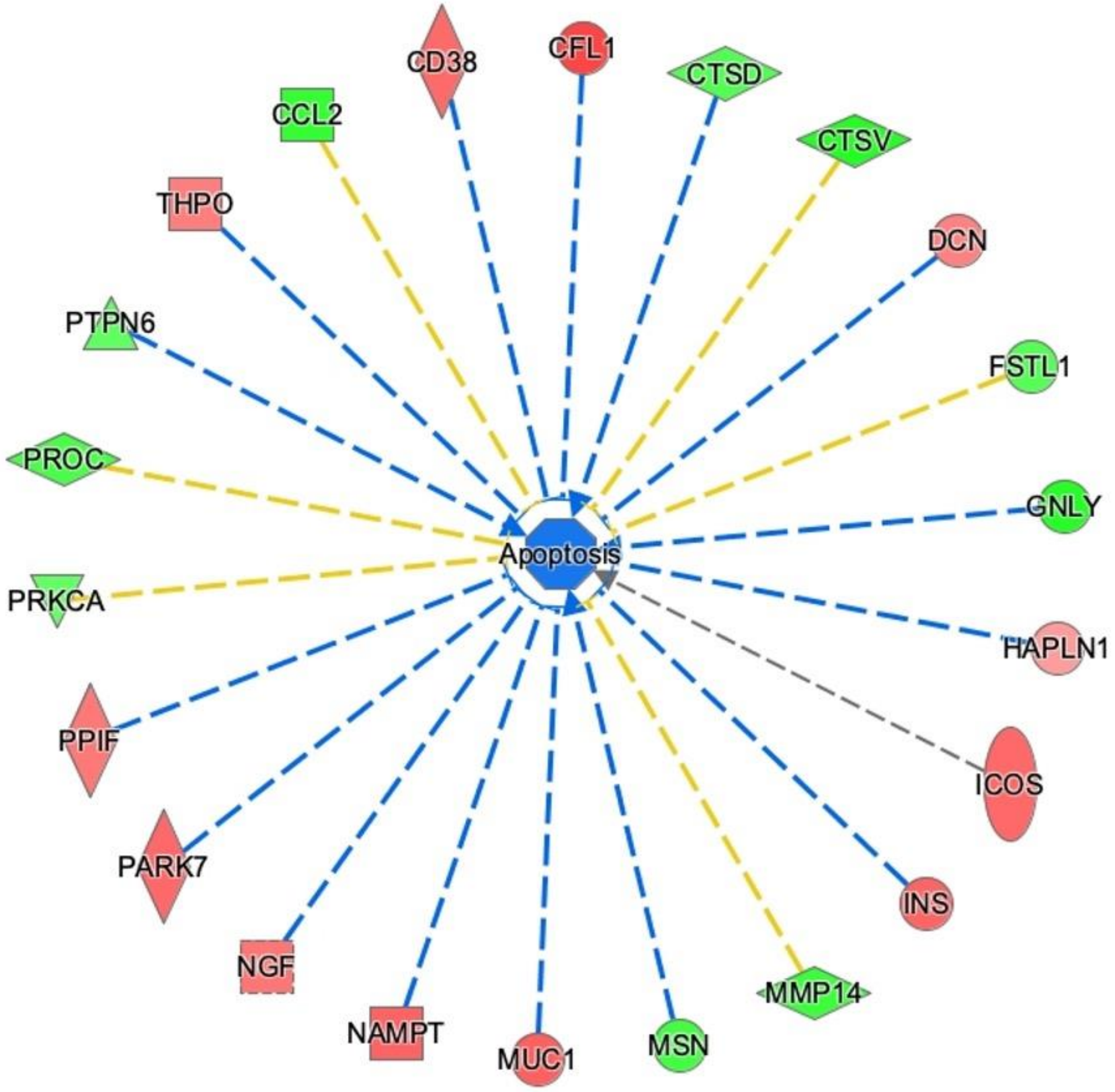
CD38
CFL1
CTSD
CCL2
CTSV
THPO
DCN
PTPN6
FSTL1
PROC
GNLY
Apoptosis
PRKCA
HAPLN1
PPIF
ICOS
PARK7
INS
NGF
MMP14
NAMPT
MUC1
MSN

D. Long-term synaptic depression

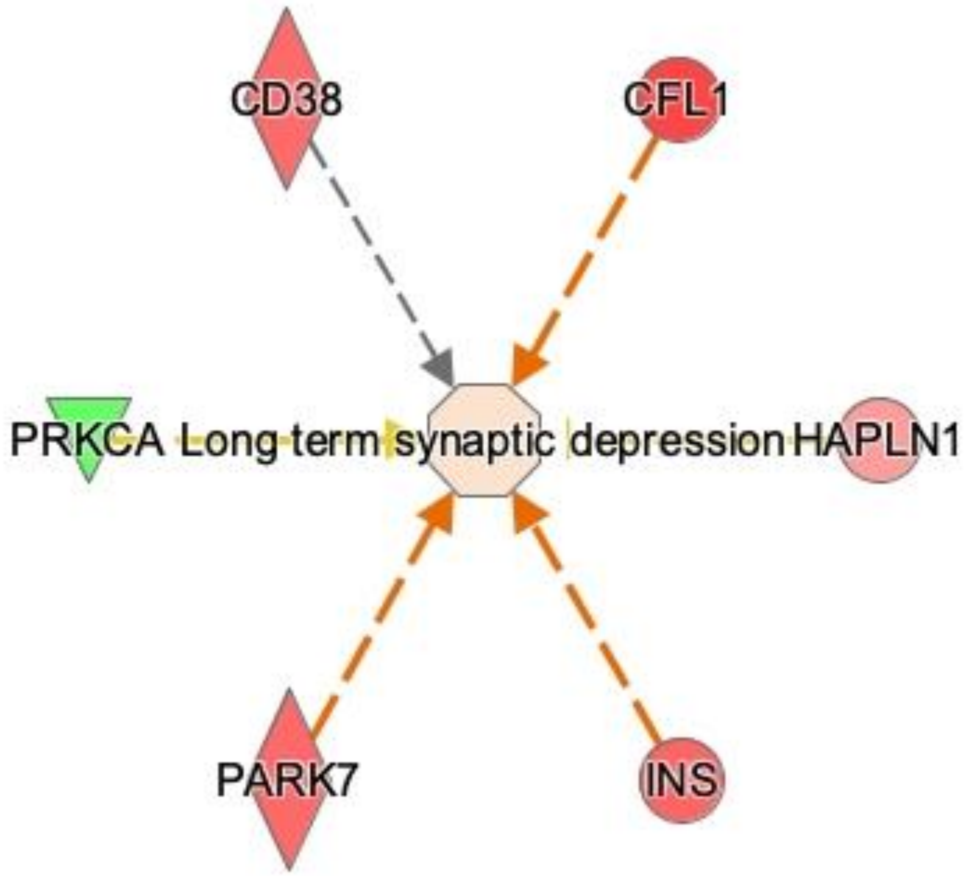

E. Angiogenesis

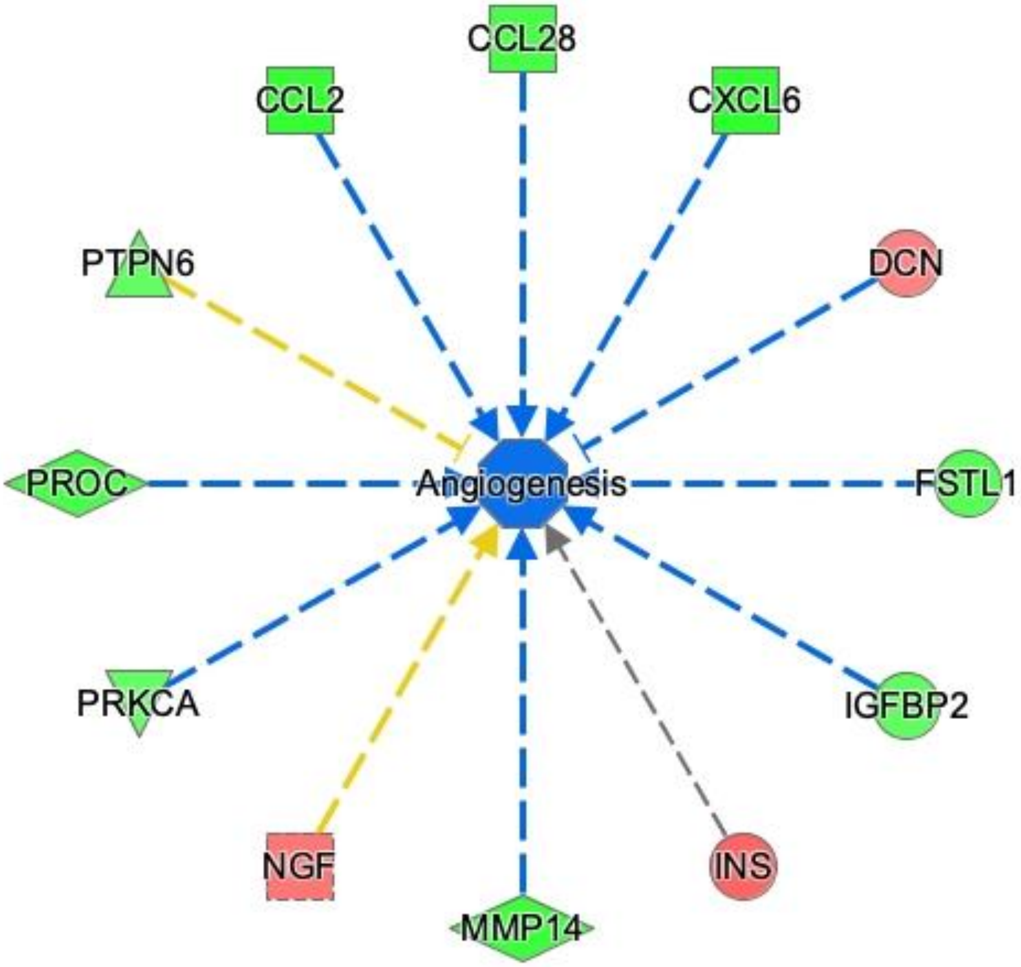

F. Neuronal cell death

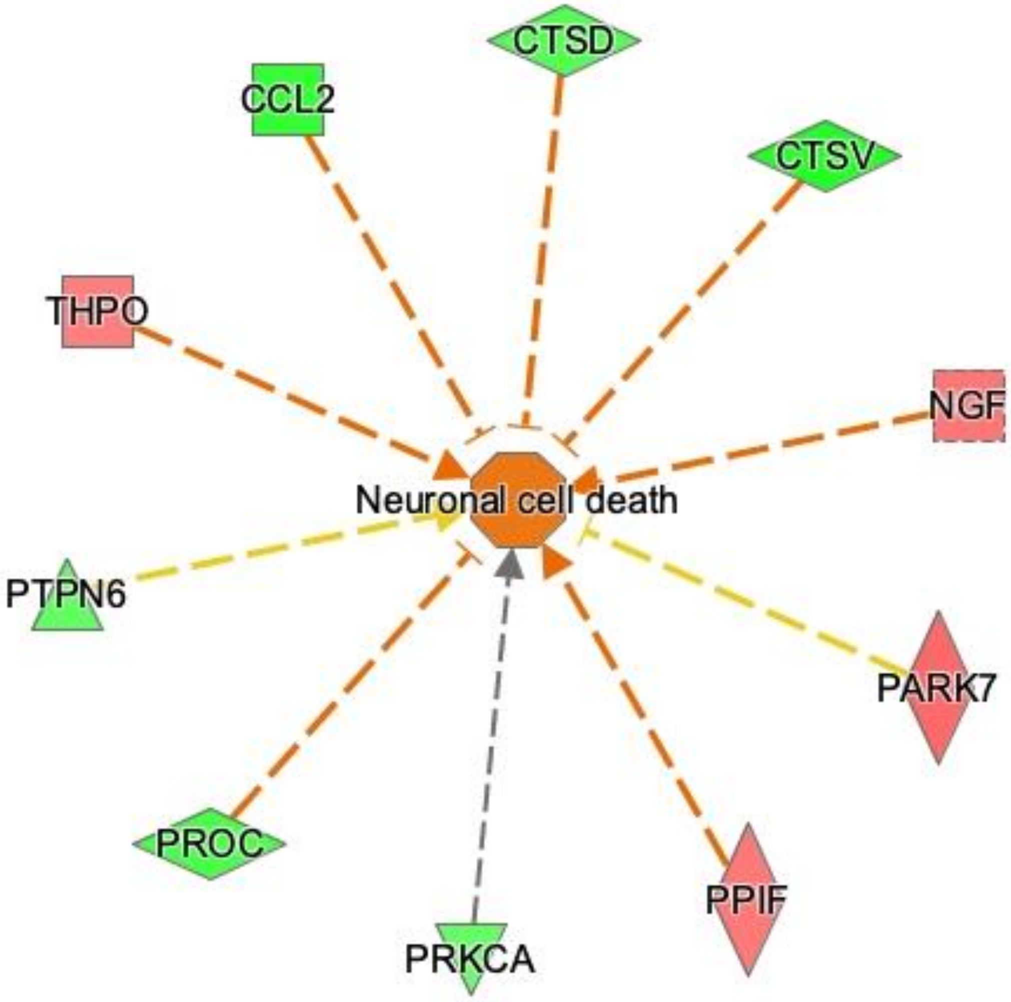

Supplementary Figure S2: Upstream Regulator Analysis of the 32 Significant CSF Proteins

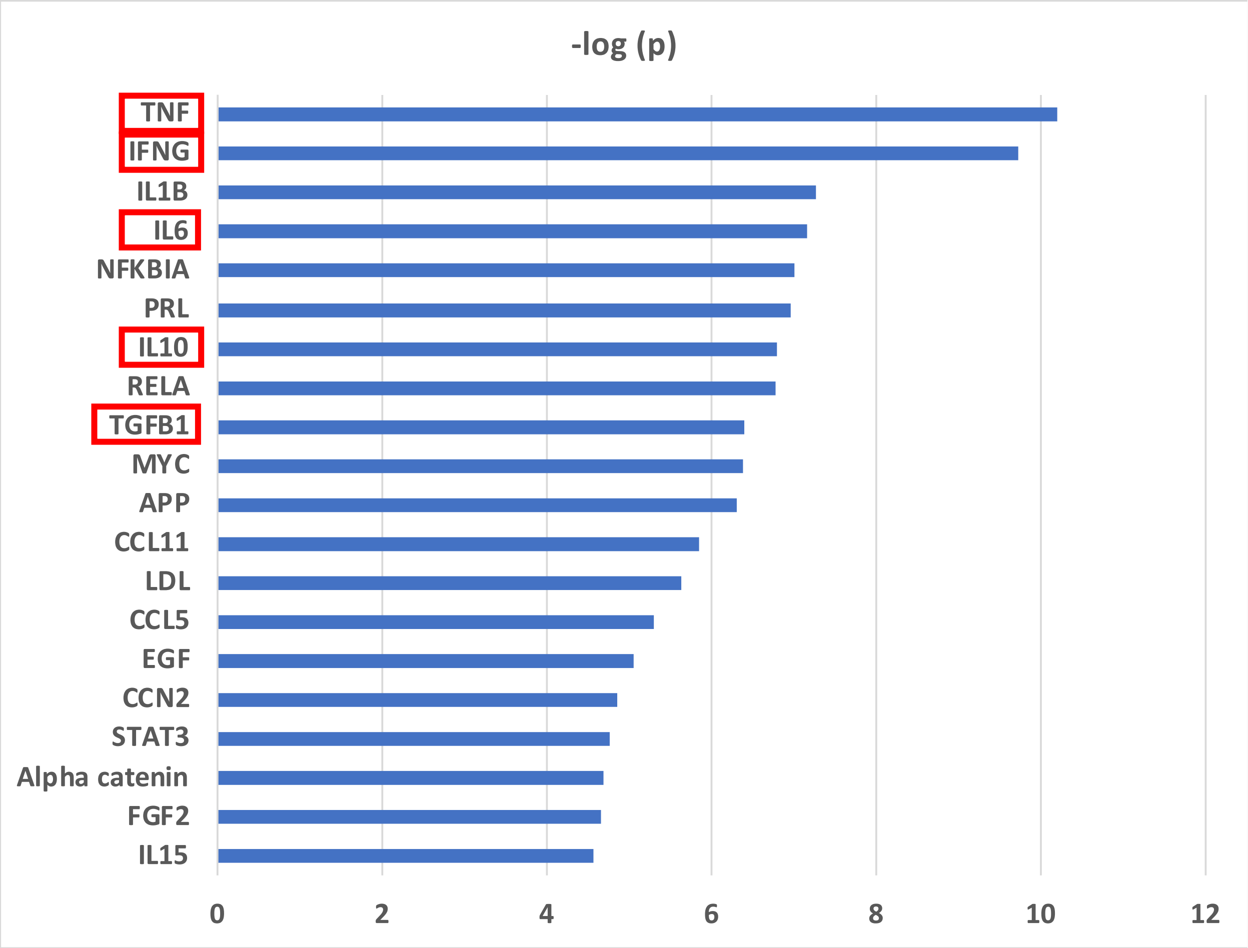

Supplementary Figure S3: Proteins Differentially Expressed in Delirium for Cytokine and Chemokine Signaling Pathways

A. Cytokine Tumor Necrosis Factor-α

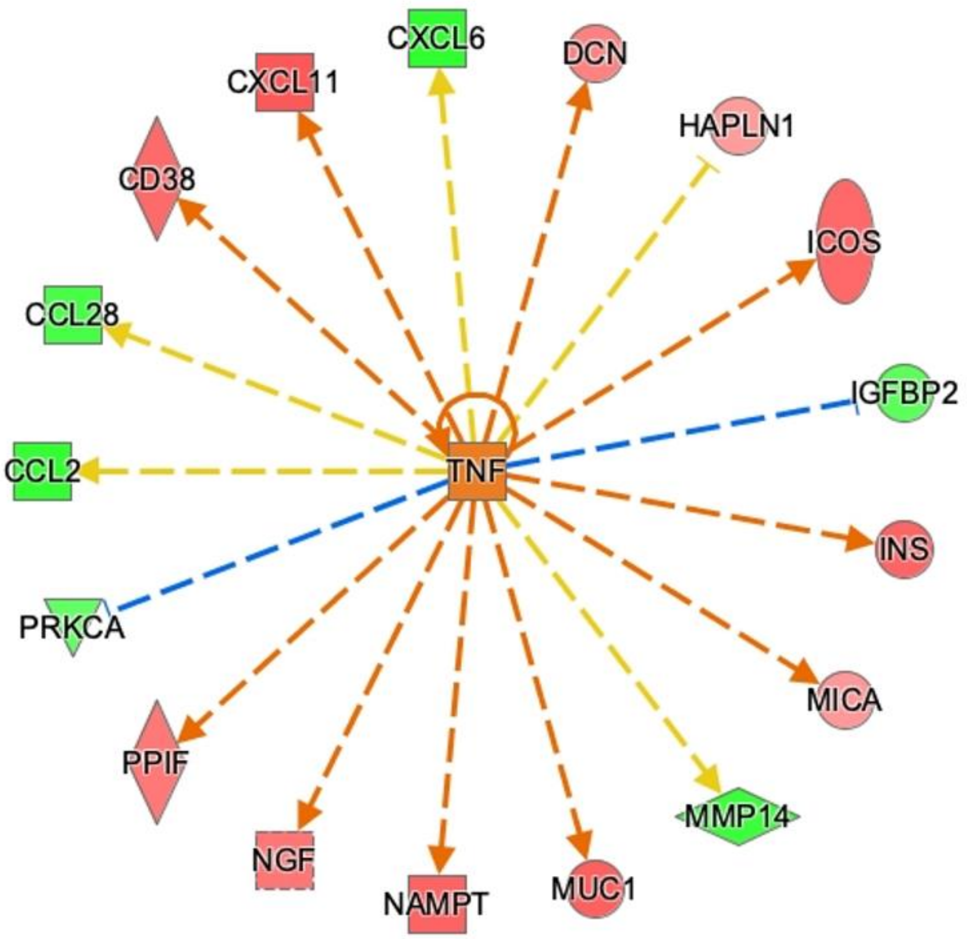

B. Cytokine Interferon-γ

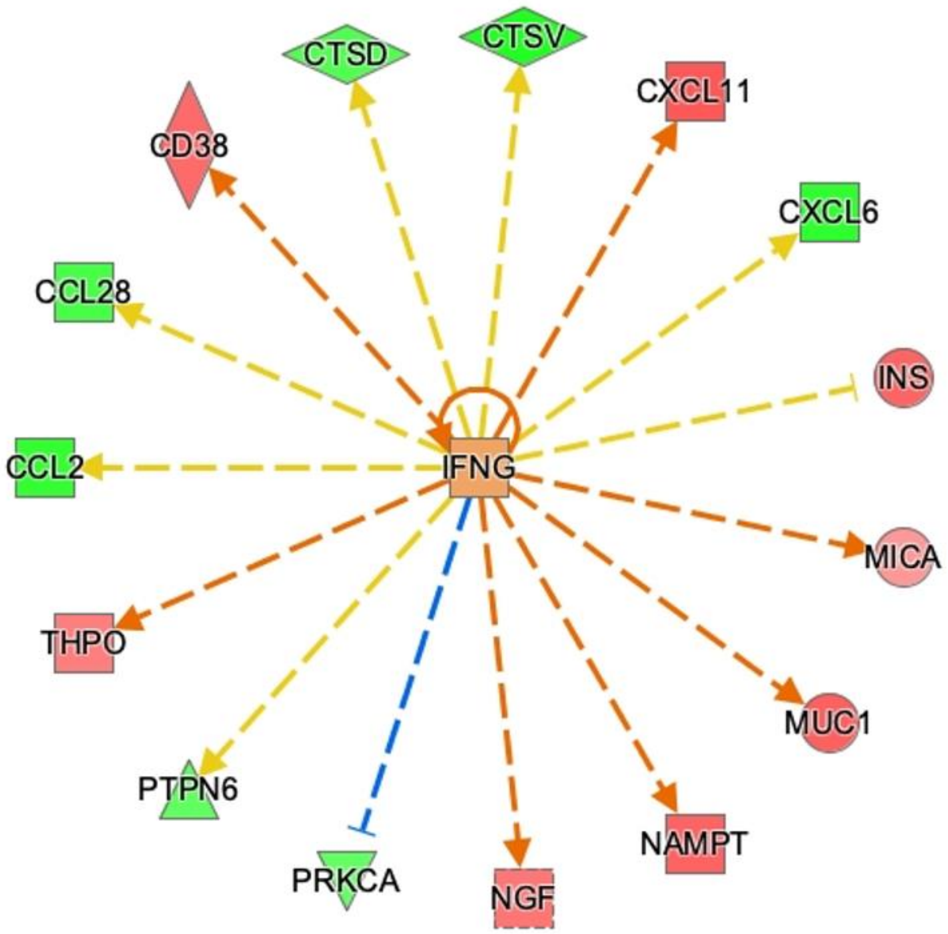

C. **Cytokine Interleukin-6**

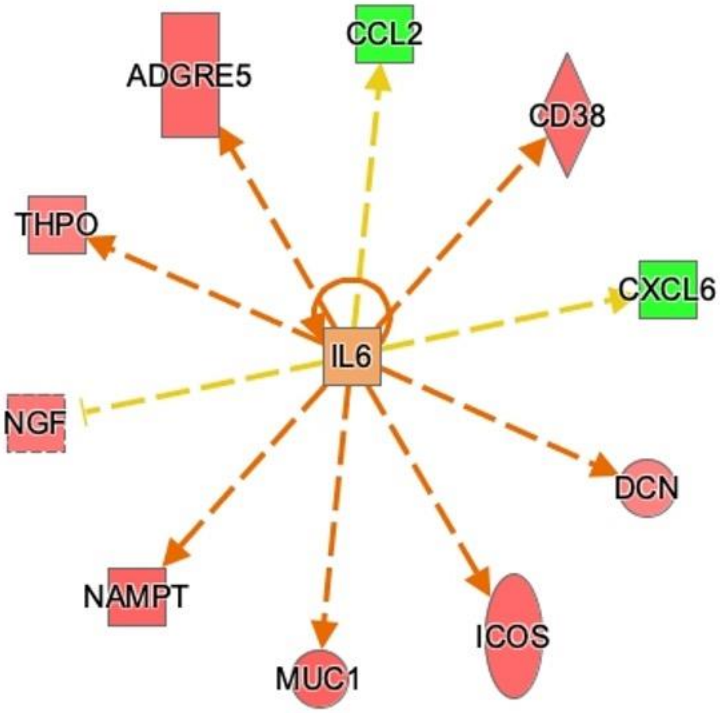

D. Cytokine Interleukin-10

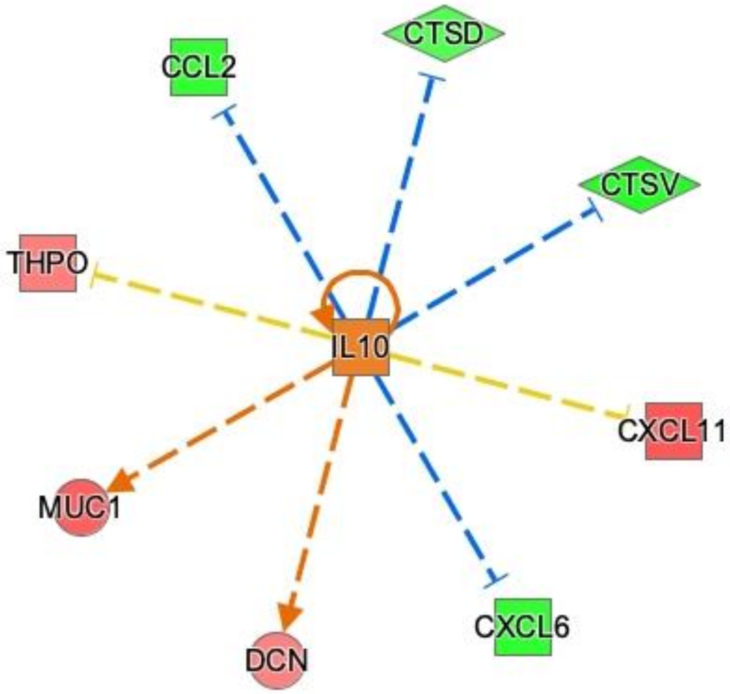

E. Cytokine Transforming Growth Factor-β1

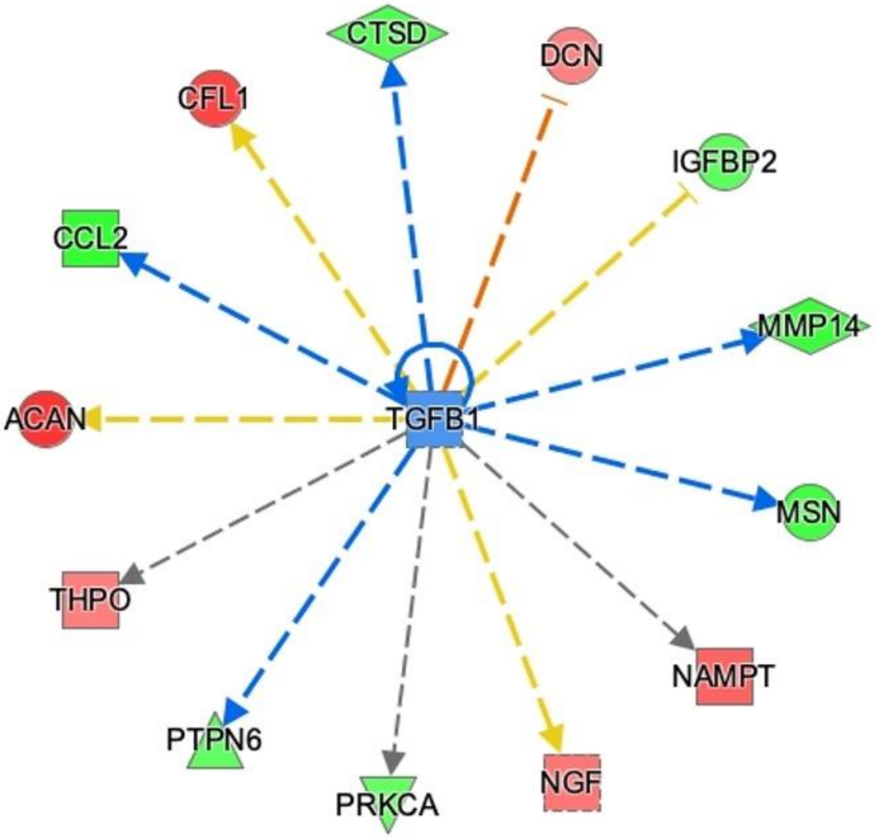

Supplementary Figure S4: Shared Overlap of Proteins for Delirium Incidence and Delirium Severity

A. **Delirium incidence t-test p-value < 0.05 and delirium severity Spearman p-value < 0.05**

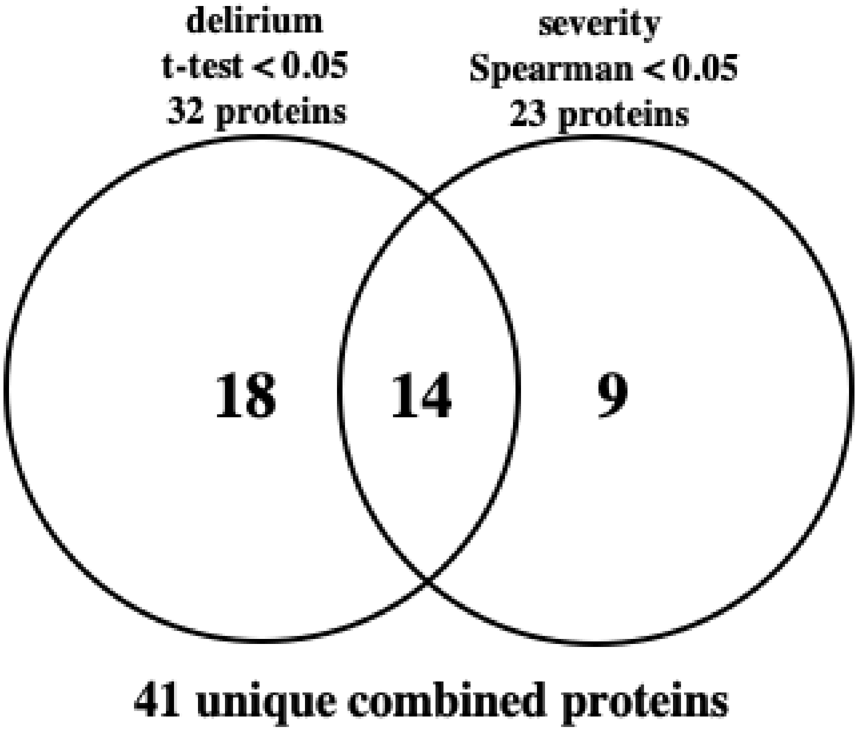

B. Delirium incidence t-test p-value < 0.10 and delirium severity Spearman p-value < 0.10

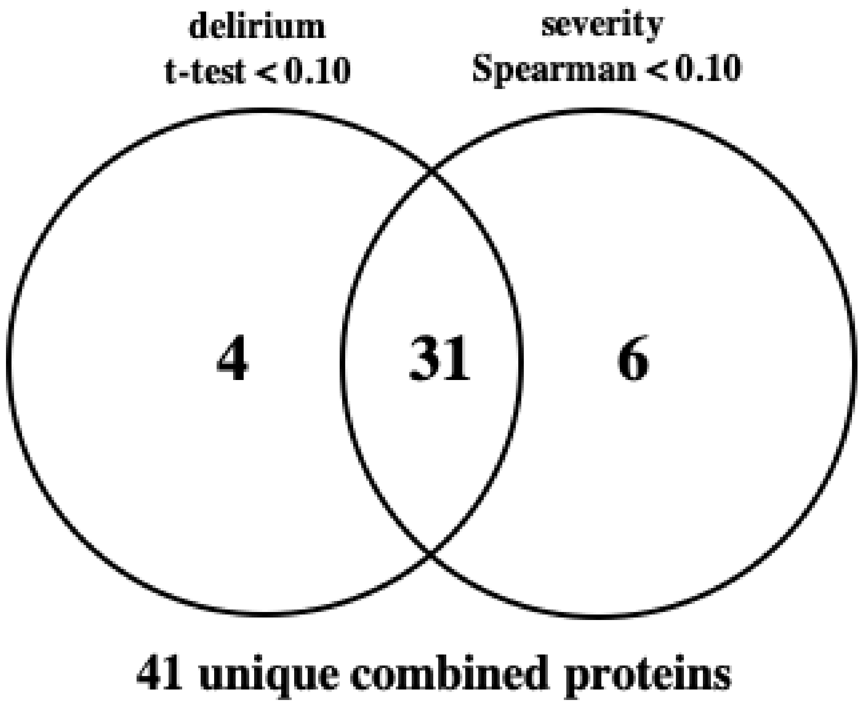

Supplement: Supplementary file 1 [file biomolecules-13-01395-s001.zip › biomolecules-2578126-supplementary.pdf]
